# Single-cell RNA-seq revealing the immune features of donor liver during liver transplantation

**DOI:** 10.3389/fimmu.2023.1096733

**Published:** 2023-02-08

**Authors:** Yi Shan, Debin Qi, Lei Zhang, Lixue Wu, Wenfang Li, Hao Liu, Tao Li, Zhiren Fu, Haili Bao, Shaohua Song

**Affiliations:** ^1^ Department of Emergency and Intensive Care Unit, Shanghai Changzheng Hospital, Naval Military Medical University, Shanghai, China; ^2^ Department of General Surgery, Ruijin Hospital, School of Medicine, Shanghai Jiaotong University, Shanghai, China

**Keywords:** single cell RNA sequence, liver transplantation, donor liver, immune repertoire, transcriptome

## Abstract

Immune cells, including T and B cells, are key factors in the success of liver transplantation. And the repertoire of T cells and B cells plays an essential function in mechanism of the immune response associated with organ transplantation. An exploration of their expression and distribution in donor organs could contribute to a better understanding of the altered immune microenvironment in grafts. In this study, using single-cell 5’ RNA sequence and single-cell T cell receptor (TCR)/B cell receptor (BCR) repertoire sequence, we profiled immune cells and TCR/BCR repertoire in three pairs of donor livers pre- and post-transplantation. By annotating different immune cell types, we investigated the functional properties of monocytes/Kupffer cells, T cells and B cells in grafts. Bioinformatic characterization of differentially expressed genes (DEGs) between the transcriptomes of these cell subclusters were performed to explore the role of immune cells in inflammatory response or rejection. In addition, we also observed shifts in TCR/BCR repertoire after transplantation. In conclusion, we profiled the immune cell transcriptomics and TCR/BCR immune repertoire of liver grafts during transplantation, which may offer novel strategies for monitoring recipient immune function and treatment of rejection after liver transplantation.

## 1 Introduction

Liver transplantation is the only option available for the treatment of end-stage liver disease(1). But the development of liver transplantation surgery is restricted by numerous immunological factors, including liver ischemia-reperfusion injury (IRI)(2, 3), graft-related rejection(4) and graft-versus-host disease (GVHD)(5). These all poses a great challenge for the prognosis of recipients. The mechanisms underlying all these pathological processes are not fully understood, and generally acknowledged that they are the result of the interaction of a diverse range of cells, especially immune cells, and various inflammatory mediators and cytokines(6–8). From the cold storage to the implantation in recipients, the donor livers are subjected to a complex pathophysiological process(9). Different immune cells, or even the same type of cells in distinct microenvironments, will have different roles during the course of IRI, immune rejection or GVDH(2, 10, 11). Enhancing our understanding of the complex immune microenvironment during liver transplantation could greatly contribute to immune research and treatment related to liver transplantation.

A large quantity of heterogeneous parenchymal and mesenchymal cells makes up the liver, which includes a variety of immune cell subsets with different functions, such as macrophages, T cells, and B/plasma cells(12). Single-cell RNA sequencing (scRNA-seq) technique has allowed us to comprehensively resolve liver ecosystems and understand the cellular heterogeneity(13). The scRNA-seq has been applied in many diseases to investigate the immune cell populations(14–16). Therefore, we performed a full-scale scRNA-seq of intrahepatic immune cells during transplantation to study the altered immune microenvironment during liver transplantation.

T cell receptor (TCR) and B cell receptor (BCR) act as critical molecules in adaptive immunity and determine most of the functions of T cells and B cells(17). The diversity of TCR and BCR libraries is the result of rearrangements of variable (V), diversity (D) and linkage (J) genes at the complementary determining region 3 (CDR3) recombination junctions(18), and have been determined to be involved in autoimmune diseases(19), cancers(20), and solid organ transplantations(21). To identify graft-associated TCRs and BCRs may serve as novel biomarkers that could provide new insights into the immune status and therapeutic strategies after liver transplantation.

Herein, we collected three pairs of pre- and post-transplantation human donor liver samples and used 5’ scRNA-seq and scTCR/BCR repertoire sequencing to comprehensively profile all the intrahepatic immune cell types and their transcriptomic changes. This study can elucidate the immune status changes of the donor livers during the surgery and try to find the TCR/BCR associated with organ transplantation immunity.

## 2 Materials and methods

### 2.1 Grafts and patients information

All liver donations on record were voluntary after cardiac death. All cases were provided with their informed permission. Immunosuppression treatment and a routine pre-liver transplantation examination were given. Data related to patient and donor including gender, age, blood type, etiology and other laboratory data are presented in **Supplementary Table S1**. The study has been approved by the Ethics Committee of Ruijin Hospital affiliated to Shanghai Jiao Tong University School of Medicine.

### 2.2 Human liver sample collection and dissociation

Donor liver organs were obtained using standardized procedures, perfused, and stored in a cold (4°C) University of Wisconsin (UW) solution. “Cold ischemic time”, the period between perfusion of the graft with UW solution and removal of it from the refrigeration, was limited to 8 hours for each person. Pre-LT donor liver samples (pre group: before implantation) were taken from the left lobe of the livers during the back-table preparation, while the post-LT liver samples were taken 3 hours after portal vein reperfusion (post group: before the abdominal closure). Within 48 hours of liver transplantation surgery, the levels of alanine aminotransferase (ALT) and aspartate aminotransferase (AST) in patient’s blood were tested, which reached their peak values. Following tissue collection, 4°C physiological saline was used to rapidly remove any blood or cold UW solution from the liver tissues. Then, the tissues were cut into 1 mm^3^ pieces in 1640 medium (Meiluncell) and treated with the digesting solution (1 mg/ml Collagenase II + 1 mg/ml Collagenase IV + 50 ug/ml DNase I) for 30 minutes at 37°C into single-cell suspension. The single-cell suspension was then passed through a 70 mm nylon cell strainer before centrifuged at 300 g for 5 min to collect the cell pellet. Red blood cells were lysed using Erythrocyte Lysis Buffer. After washing twice with PBS (Meiluncell), the cells were resuspended at a cell density of 50–500 million cells per ml in resuspension buffer (PBS with 2% fetal bovine serum (Gibco)).

### 2.3 Single-cell 5’ RNA-seq data processing

ScRNA-seq libraries and scTCR/BCR repertoire were created using the 10× Genomics Chromium Single-cell 5’ Library and single cell V(D)J reagent kits according to the platform’s instructions (10× Genomics, USA). Briefly, the combination of cells, chemicals, and gel beads with barcodes wrapped in oil droplets is referred described as a “GEM”. Following the dissolution of the gel beads in the GEM, cells were lysed to liberate mRNA. Barcoded cDNA was produced using reverse transcription and utilized for sequencing. The generated cDNA was adequate to build 5’ gene expression libraries and TCR/BCR libraries. Purified 5’ libraries were sequenced by Illumina Hi-Seq XTen platform at paired-end 150-bp length. VDJ region-enriched libraries were analyzed on an Illumina HiSeq 2500 device. Cell Ranger (version 6.0.2, 10× Genomics) was applied to align the sequence and generate cell barcode matrix. For the TCR/BCR clonotype analysis, we elaborate on this in a later section.

### 2.4 Quantification and subcluster

Seurat R package (version: 4.0.3) was used for data filtering, cell normalization and clustering based on the expression table according to the unique molecular identifier (UMI) counts of each sample(22). Low quality cells will be removed: genes expressed < 500, UMI < 1000, and mitochondrial genome ratio > 0.2. Then we performed principal component analysis (PCA analysis) from scaled data, where the top 2000 high variable genes and the top 10 principal were used to construct UMAP. Differential expression markers were analyzed using the “FindAllMarkers” function and significance levels were tested using the Wilcoxon test. Marker genes for each cluster were identified by using the Wilcox rank sum test in the “FindAllMarkers” function for genes expressed in at least 10% of cells and a 0.25 mean natural log-transformed ploidy change criterion. We considered a gene to be a cluster-specific marker when it was differentially upregulated in a cluster relative to other clusters. Cell clusters were annotated as known biotypes with canonical marker genes according to Cell Marker(23) (http://bio-bigdata.hrbmu.edu.cn/CellMarker/) database and previous article(24, 25).

### 2.5 Differential expression gene analysis

To identify differentially expressed genes among samples, the function “FindMarkers” with wilcox rank sum test algorithm was used under following criteria: log2FC > 0.25, *p* value<0.05, min.pct > 0.1.

### 2.6 Functional enrichment analysis

Gene ontology (GO) analysis was performed to illustrate the biological implications of marker genes and differentially expressed genes. We downloaded the GO annotations from NCBI (http://www.ncbi.nlm.nih.gov/), UniProt (http://www.uniprot.org/) and the Gene Ontology (http://www.geneontology.org/). Fisher’s exact test was applied to identify the significant GO categories and FDR was used to correct the *P*-values. Pathway analysis was used to find out the significant pathway of the marker genes and differentially expressed genes according to KEGG database. We turn to the Fisher’s exact test to select the significant pathway, and the threshold of significance was defined by *P*-value and FDR. To characterize the relative activation of a given gene set such as pathway activation, we performed Quantitative Set Analysis of Gene Expression (QuSAGE, 2.16.1) analysis(26).

### 2.7 SCENIC analysis

To assess transcription factor regulation strength, we applied the Single-cell regulatory network inference and clustering (pySCENIC, v0.9.5) workflow, using the 20-thousand motifs database for RcisTarget and GRNboost (27).TCR/BCR Repertoire Profiling

The scTCR/BCR-seq data was manipulated with Cell Ranger (version 6.0.2, 10× Genomics) with reference to the human VDJ reference offered by 10× Genomics. Cells with at least one productive TCR α or TCR β chain were retained for further analysis, such as the diversity of TCR/BCR repertoire, the usage and combination of V(D)J gene, and the distribution of CDR3 length(28). In order to combine TCR or BCR results with the gene expression, the TCR- or BCR-based assays was conducted only for cells that were identified as T or B cells respectively. In addition, T or B cell clonotype shared among clusters or samples were identified.

### 2.8 Histological staining

For staining, liver samples were fixed overnight in 4% paraformaldehyde, embedded in paraffin wax, and serially sectioned at 4 μm thickness. At least 10 high-power fields (magnifications of 10× and 40×) per slice were evaluated for each tissue sample.

## 3 Results

### 3.1 Single cell atlas construction of human donor liver during transplantation

**Figure 1:**
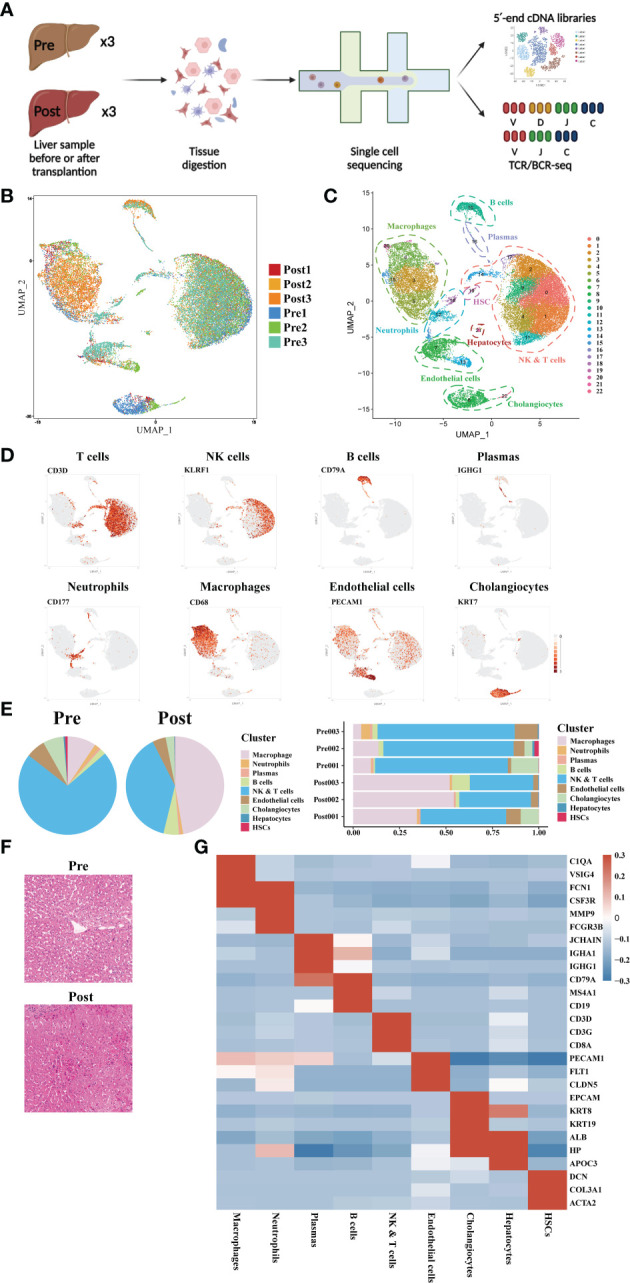
Single-cell atlas of human donor liver in transplantation surgery. **(A)** Schematic diagram of scRNA-seq and TCR/BCR repertoire sequencing workflow. **(B)** Distribution of the 6 liver samples on UMAP plots. **(C)** UMAP plots for the identification of 22 cell clusters for all cells. **(D)** Expression of canonical cell markers including CD3D, KLRF1, CD79A, IGHG1, CD177, CD68, PECAM1, and KRT7. **(E)** Cell proportion in different liver samples. **(F)** H&E staining of donor liver samples pre- and post- transplantation. **(G)** Heatmap of top genes of different cell clusters.

Three pairs of donor liver samples from three adult males were performed scRNA-seq analysis. A total of 39,505 cells transcriptomes from these 6 samples were captured using 10× Genomics platform, and the medians for UMI and genes were 2812-3672 and 1260-1629 respectively (**Figure 1A**). After quality control (QC) filtration, transcriptional information from a total of 28985 cells was eventually available for further analysis. We conducted dimensional reduction and clustering of the obtained intrahepatic cells based on UMAP. All these cells were annotated into 22 clusters using canonical marker genes, and each individual cluster consisted of a proportionate number of cells from each sample (**Figure 1B**). These clusters across 8 major cell lineages, including NK cells, T cells, B cells, plasmas, monocytes, neutrophils, cholangiocytes, and endothelial cells (**Figure 1C**). Each type of cell was identified by their canonical gene marker profiles such as T cell (CD3D(29)), NK cell (KLRF1(30)), B cell (CD79A(31)), plasmas (IGHG1(31)), neutrophils (CD177(32)), macrophages (CD68(33)), endothelial cells (FCN2(33)), and cholangiocytes (KRT7(34)), respectively (**Figure 1D** and **Supplementary Table S1**). From the distribution of cellar fractions, it was clear that T cells, NK cells and macrophages prevailed the liver transplantation in each sample (**Figure 1E**). It was apparent that the proportions of macrophages were higher in the post liver samples, and the difference was also present in the proportions of NK and T cells (**Figure 1E**). This result suggested that there was a significant difference in the detailed structure of immune cell compartments during liver transplantation. The clinical data of the samples and liver H&E staining are shown in **Supplementary Table S1** and **Figure 1F**. And we can tell from the image of H&E staining that there was a certain degree of destruction of the liver tissue structure after reperfusion. The differential gene expression of each cell cluster is shown in **Figure 1G**.

### 3.2 scRNA-seq of macrophage clustering and subtype analysis during liver transplantation

**Figure 2:**
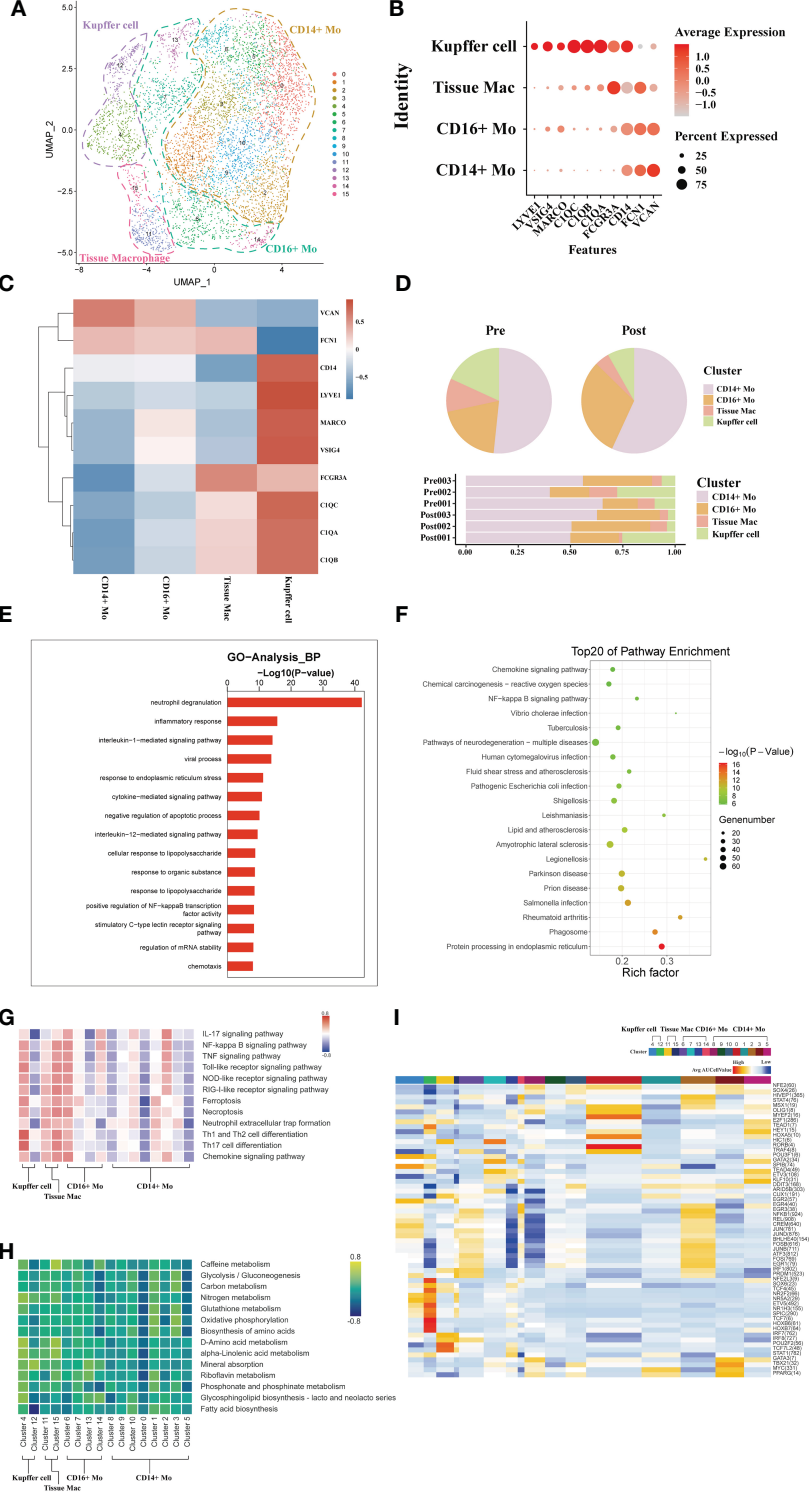
scRNA-seq of macrophages during liver transplantation. **(A)** UMAP plotting of 16 clusters of mononuclear phagocytes and divided into 4 different cell subpopulations. **(B)** Expression of VSIG4, C1QC, IL1B, VCAN, CD14, and FCGR3A genes for monocytes and Kupffer cells clusters. **(C)** Heatmap of 3 differentially expressed genes of each monocytic/Kupffer cell clusters. **(D)** Pie plot and bar plot showing the proportion of monocytic/Kupffer cells in each sample. **(E)** Bar plot showing GO analysis results in different samples of monocytic/Kupffer cells during the transplantation. **(F)** Bubble plot showing the top 20 pathways enrichment in different samples of monocytic/Kupffer cells during the transplantation. **(G)** Result of QuSAGE analysis showing the selected pathways activation in different monocytic/Kupffer clusters. **(H)** Heatmap of selected metabolism pathways activation in different monocytic/Kupffer clusters. **(I)** Heatmap of SCENIC analysis in different monocytic/Kupffer clusters.

The liver is a solid organ in the human body with the largest number of tissue resident macrophages. In order to investigate the dynamics of different macrophages subtypes during liver transplantation, a total of 6207 macrophages obtained from three pairs of donor samples were divided into four subsets based on the expression of typical monocytic/Kupffer cell marker genes. Based on the specific high expression of genes in cell subsets, we identified two monocyte subsets (CD14+ and CD16+ Monocytes), a tissue macrophage subset (highly expressed FCN1 and FCGR3A gene) and a Kupffer cell subset (highly expressed C1QC) (**Figure 2A**).

The CD14+ and CD16+ monocytic cell clusters highly express S100A8 and other similar genes to those expressed by peripheral mononuclear cells, suggesting that these two clusters may have been recruited and differentiated from peripheral blood circulating mononuclear cells. High expression of C1QC, C1QB and other tissue-resident marker genes in the Kupffer cell and inflammatory macrophage clusters suggests that they are the liver tissue-resident macrophages (**Figures 2B, C**). To further explore the dynamics of different monocytic and Kupffer cell clusters during liver transplantation, a comparative analysis of pre- and post-operative samples was carried out. In the post- samples, the proportion of Kupffer clusters was lower than that in the pre- samples, while the proportion of monocyte clusters was higher than that in the pre- samples (**Figure 2D**).

The liver is an immune tolerant organ and Kupffer cells are the most important component in limiting the activation of inflammation in the liver. Subsequently, we performed the GO analysis of differential genes in Kupffer cells of the donor liver pre- and post- transplantation to analyze the differences in biological function of Kupffer cell. The Kupffer cell clusters were found to have increased expression of *neutrophil degranulation*, *inflammatory response* genes, and *immune response* that promote inflammatory function after transplantation (**Figure 2E**). KEGG pathway analysis also indicated activation of inflammation-related pathways in Kupffer cells (**Figure 2F**). The results of GO and KEGG pathway analysis confirmed that the altered immunosuppressive environment was closely associated with the rapid activation of inflammatory pathways in Kupffer cells in the liver.

To further explore differences in the variation of functional pathways associated with different subpopulations of monocytic/Kupffer cells, we performed QuSAGE analysis Two different phenotypes of Kupffer cell clusters were identified (**Figure 2H**). Analyzed with selected pathways related to inflammation, the results show that cell death pathways (such as *Ferroptosis, NOD-like receptor signaling pathway*), inflammation-related pathways (*IL-17 signaling pathway* and *TNF signaling pathway*) of cluster 4 of Kupffer cells were significantly activated (**Figure 2I**). And the cluster 4 of Kupffer cells also play a role in promoting the activation of a wide range of immune cells (such as *Th1 and Th2 cell differentiation, and Th17 cell differentiation*). Most of the pathways enriched in the tissue macrophage clusters are the same as those in the Kupffer clusters. But the tissue macrophage clusters play more of a role in promoting the activation of inflammation. In common with Kupffer cell cluster, CD14 + and CD16+ monocyte clusters also exhibited activation of multiple pro-inflammatory pathways (**Figure 2G**). However, Kupffer cells exhibit a stronger pro-inflammatory effect. Different monocytic/Kupffer cell subpopulations also showed marked differences in the outcome of metabolic pathway enrichment. Kupffer clusters are more activated in *glycolysis and amino metabolism*-related pathways, while monocytic clusters exhibit enrichment in *oxidative phosphorylation* related metabolic pathways (**Figure 2H**). The result of SCENIC analysis shows the transcription factor activation differs markedly between macrophage types. The cluster 4 of Kupffer cells exhibits significant activation of the NR2F2, HOXB transcription factor, which may be closely related to its function in suppressing the inflammatory response (**Figure 2I**).

### 3.3 scRNA-seq of CD4+T cell clustering and subtype analysis during liver transplantation

**Figure 3:**
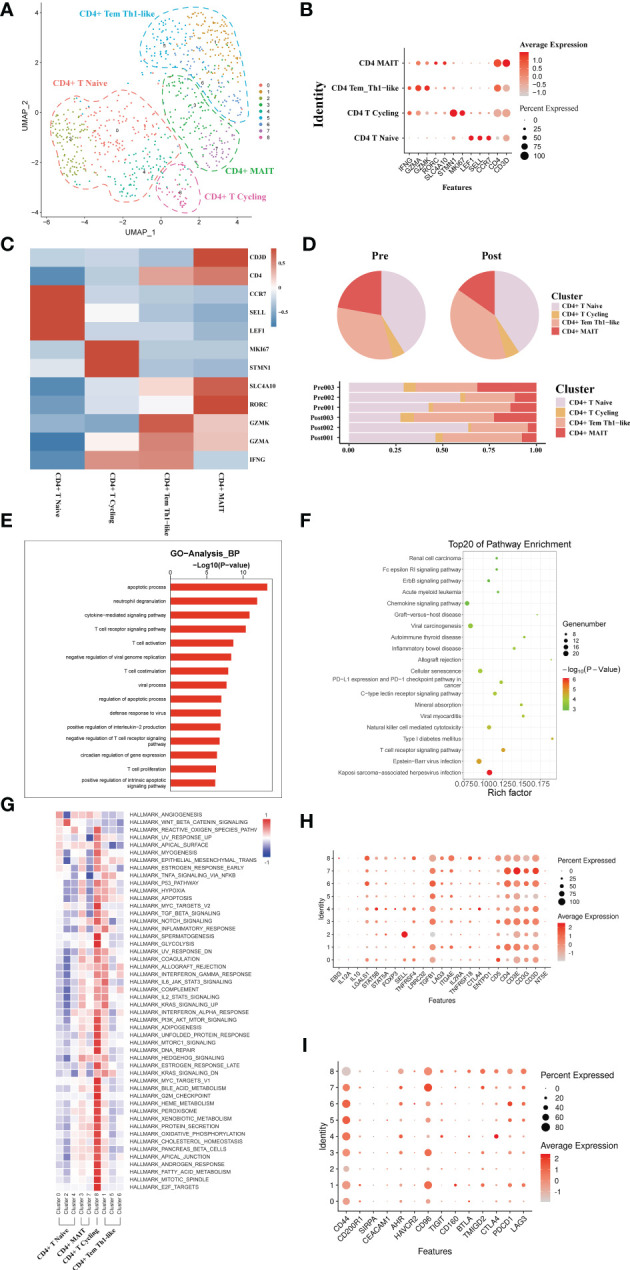
scRNA-seq of CD4+ cell clustering and subtype during liver transplantation. **(A)** UMAP plotting of 8 clusters of CD4+ T cells and divided into 4 different cell subpopulations. **(B)** Expression of CCR7, GZMA, MKI67, and SLC4A10 genes for CD4+ T cell clusters. **(C)** Heatmap of 3 differentially expressed genes of each CD4+ T cell cluster. **(D)** Pieplot and barplot showing the proportion of CD4+ T cell clusters in each sample. **(E)** Barplot showing GO analysis results of CD4+ T cell clusters. **(F)** Result of KEGG pathway analysis showing the top 20 significantly different pathways activation in different samples of CD4+T cell clusters. **(G)** Heatmap of hallmark pathways activation in different CD4+T cell clusters. **(H)** Expression of Treg related genes expressed in CD4+ T cells. **(I)** Expression of immune checkpoint inhibition receptor genes expressed in CD4+ T cells.

A total of 793 CD4+ T single cell transcriptomes were obtained from three pairs of liver samples and reconstituted into 9 clusters (**Figure 3A**). Based on typical genetic markers, we grouped these nine cell clusters into four CD4+ T cell lineages. The cells were annotated as CD4+ memory T cells (Tem, GZMK), CD4+ Naive T cells (CCR7, LEF1), CD4+ mucosa-associated invariant T cells (MAIT, SLC4A10), and CD4+ T cycling cells (MKI67) based on their characteristic expression profiles (**Figures 3B, C**). To investigate the dynamics of CD4+ T cell clusters during liver transplantation, we found that the proportion of CD4+ MAIT decreased post-operatively, while the proportion of CD4+Tem cell clusters increased (**Figure 3D**). GO analysis showed that CD4+ T cells in post-operative grafts were enriched in biological processes of *apoptosis*, inflammatory cell activation (*cytokine−mediated signaling pathway*) and adaptive immune response (*T cell activation*) (**Figure 3E**). GO analysis of DEG in pre- and post-operative samples showed that CD4+ T cell clusters were activated in the *Chemokine signaling pathway*, *Graft−versus−host disease*, *Allograft rejection*, *T cell receptor signaling pathway* and other pathways associated with inflammation and lymphocyte activation during the surgery (**Figure 3F**). QuSAGE was used to observe the dynamic changes in specific cell clusters. We found that inflammation-related pathways such as *P53 pathway*, *inflammatory response signaling pathway*, *IL−6_JAK_STAT3 signaling pathway*, and *IL2_STAT5 signaling pathway* were significantly activated after transplantation in CD4+ Tem and CD4+ T Cycling populations (**Figure 3G**). The results of the enrichment of Treg gene and immune checkpoint receptors suggest that CD4+ Naïve T cells have the potential to differentiate into Treg cells and that activation of the immune checkpoint receptor gene in Tem cells may be able to exert immunomodulatory effects in the graft (**Figures 3H, I**)

### 3.4 scRNA-seq of CD8+ T cell clustering and subtype analysis during liver transplantation

**Figure 4:**
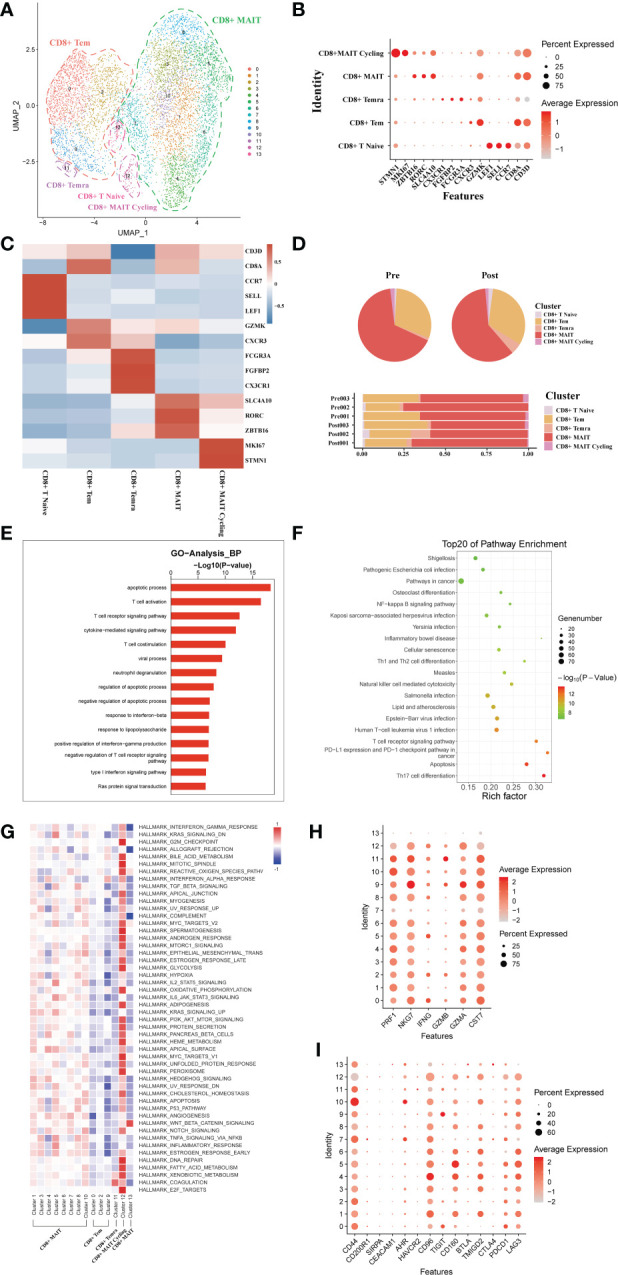
scRNA-seq of CD8+ cell clustering and subtype during liver transplantation. **(A)** UMAP plotting of 14 clusters of CD8+ T cells and divided into 5 different cell subpopulations. **(B)** Expression of CD8A, CXCR3, SLC4A10, CX3CR1, GZMK and MKI67 genes for CD8+ T cell clusters. **(C)** Heatmap of 3 differentially expressed genes of each CD8+ T cell cluster. **(D)** Pieplot and barplot showing the proportion of CD8+ T cell clusters in each sample. **(E)** Barplot showing GO analysis results of CD8+ T cell clusters. **(F)** Result of KEGG pathway analysis showing the top 20 significantly different pathways activation in different samples of CD8+T cell clusters. **(G)** Heatmap of hallmark pathways activation in different CD8+T cell clusters. **(H)** Expression of cytotoxic related genes expressed in CD8+ T cells. **(I)** Expression of immune checkpoint inhibition receptor genes expressed in CD4+ T cells.

A total of 7271 single CD8+ T cell transcriptomes were obtained from three pairs of liver samples and reconstituted into 14 cell clusters (**Figure 4A**). In accordance with previous studies, these 14 cell clusters were grouped by characteristic expression profiles into four CD8+ T cell lineages, including a CD8+ MAIT (SLC4A10, RORC), a CD8+ Tem (GZMK, CXCR3), CD8+Naïve T cell (CCR7, LEF1) and a CD8+ Temra (CX3CR1) (**Figures 4B, C**). To study the dynamics of the CD8+ T cell ratio during liver transplantation, we found an increase in the proportion of CD8+ Tem cells and a decrease in the number of CD8+ MAIT cells (**Figure 4D**). GO analysis showed that, like CD4+T cells, CD8+ T cells were also predominantly enriched in apoptosis and T cell activation functions (**Figure 4E**). GO pathway enrichment indicated that most CD8+ T cell clusters were activated in the *NF−kappa B signaling pathway* and *Natural killer cell mediated cytotoxicity* (**Figure 4F**). Observation of pathway enrichment of specific cell clusters by QuSAGE. We found that inflammatory and cell death-related pathways, such as *IL2 STAT5 Signaling*, *TNFA signaling via NFKB* and *inflammatory response pathway*, were significantly activated in the CD8+ MAIT cell clusters (**Figure 4G**). The enrichment results of metabolism-related pathways showed that CD8+ T Cycling cluster was abnormally active (**Figure 4G**). The cytotoxicity gene enrichment results showed that almost all CD8+ T cell clusters, except CD8+ Naive T cells, exhibited some cytotoxicity (**Figure 4H**). The immune checkpoint receptor gene enrichment results suggest that LAG3, CD160 and TMIGD2 may serve as effective immune checkpoint targets (**Figure 4I**).

### 3.5 Usage of V(D)J genes and frequency of clonotypes in T cells

**Figure 5:**
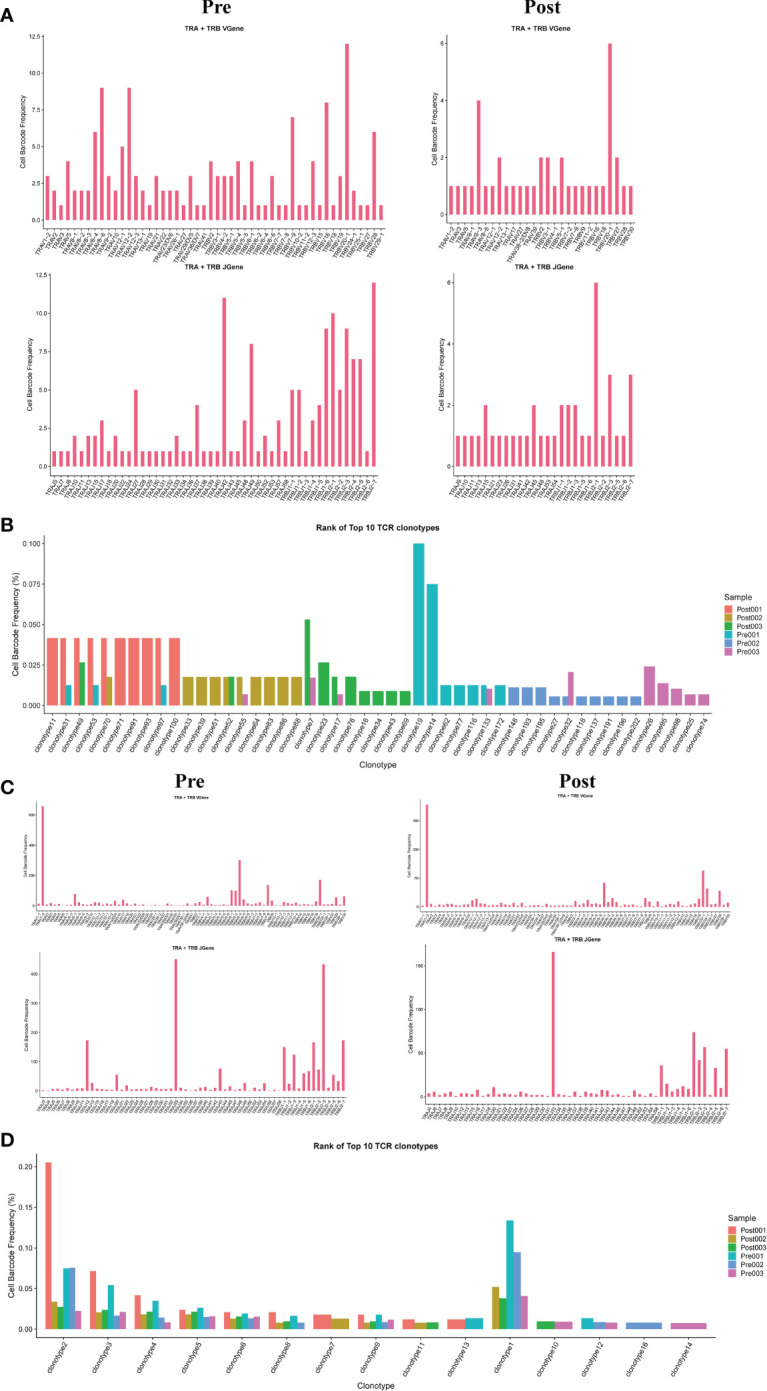
Usage of V(D)J genes and frequency of clonotypes in T cells. **(A)** The usage of the V(D)J gene of TCR α and β chain in CD4+T cell between pre and post group. **(B)** The frequency of TCR clonotypes in CD4+T cells of different groups. **(C)** The usage of the V(D)J gene of TCR α and β chain in CD8+T cells between pre and post group. **(D)** The frequency of TCR clonotypes in CD8+T cells of different groups.

The results showed that the TCR clonotypes in donor liver was more significantly reduced in CD4+T cell after transplantation, compared to CD8+T cell. (**Figures 5B, D**). In addition, the usage of VDJ genes in TCR α and β chains was biased between the pre and post group both in CD4+ and CD8+ T cells. In TCRα CD4+T cell, the most used V gene was TRAV8-6 in pre group, while in post group it is TRAV8-3 (**Figure 5A**). However, the differences of usage bias in CD8+ T cells were not as significant as those in CD4+T cells (**Figure 5C**). The overlapping of TCR among samples were shown in **Figures S1A, B**.

### 3.6 scRNA-seq of B/plasma cell clustering and subtype analysis during liver transplantation

**Figure 6:**
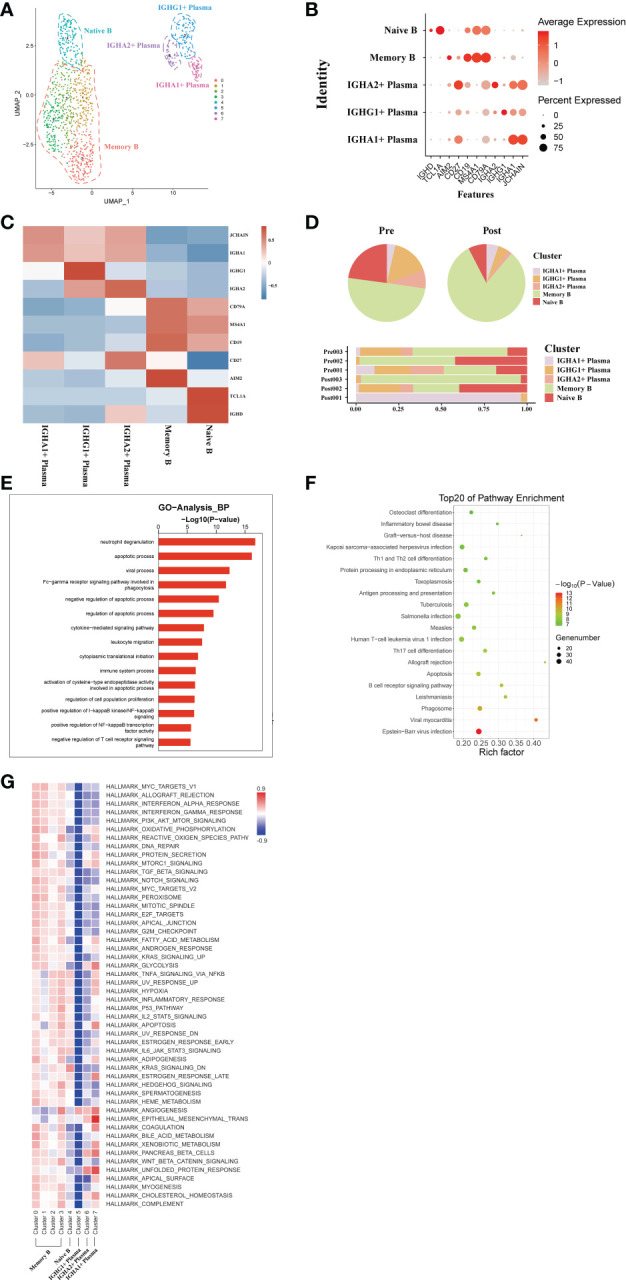
scRNA-seq of B/plasma cell clustering and subtype analysis during liver transplantation. **(A)** UMAP plot of 5 different B/plasma cell clusters during liver transplantation, colored according to diverse clusters. **(B)** Distinctive genes expression of CD79A, AIM2, TCL1A, MS4A1, IGHG1 and IGHA1 for B/plasma cell clusters. **(C)** Heatmap of 3 differentially expressed genes of each B/plasma cell cluster. **(D)** The proportion of cells in different B/plasma cell populations in each sample. **(E)** Gene Ontology enrichment analysis results of B/plasma cell clusters. **(F)** Result of KEGG pathway analysis showing the top 20 significantly different pathways activation in different samples of B/plasma cell clusters. **(G)** Heatmap of hallmark pathways activation in different B/plasma cell clusters.

A total of transcriptome information for 1026 B cells were obtained from these three pairs of donor liver samples. To investigate the dynamics of the different B subtypes, we divided B cells into six subsets based on the expression of typical B cell markers (**Figure 6A**). These clusters revealed one naive B cell (MS4A1, TCL1A) subset clusters, one memory B cell (AIM2) subset clusters, and three plasma cell (IGHA) clusters in the donor liver with different canonical marker genes (**Figures 6B, C**). Notably, the proportion of memory B cell subpopulations in the liver was significantly increased after transplantation compared to the pre-operative period, while the proportion of plasma cell clusters were decreased (**Figure 6D**). To further investigate the differential transcriptomic changes in B/plasma cells, we compared the expression profiles of B/plasma cells pre- and post- transplantation by using GO analysis. We observed an enrichment of DEG in the *neutrophil degranulation* and *apoptotic process* (**Figure 6E**). And the pathway enrichment result showed that two pathways, *Graft−versus−host disease* and *Th1 and Th2 cell differentiation* are associated with B/plasma cells (**Figure 6F**). The results of QuSAGE analysis showed that the expression of genes in the plasma cell clusters related to *Protein secretion*, while the B cell clusters related to *interferon response* pathways (**Figure 6G**). Metabolism-related pathway enrichment results suggest that plasma cell clusters exhibit enrichment in the *glycolysis* and *oxidative phosphorylation metabolism*. In contrast, B cell clusters show more enrichment in *fatty acid metabolism* and *oxidative phosphorylation metabolism* pathways (**Figure 6G**).

### 3.7 Usage of V(D)J genes and frequency of clonotypes in B cells

**Figure 7:**
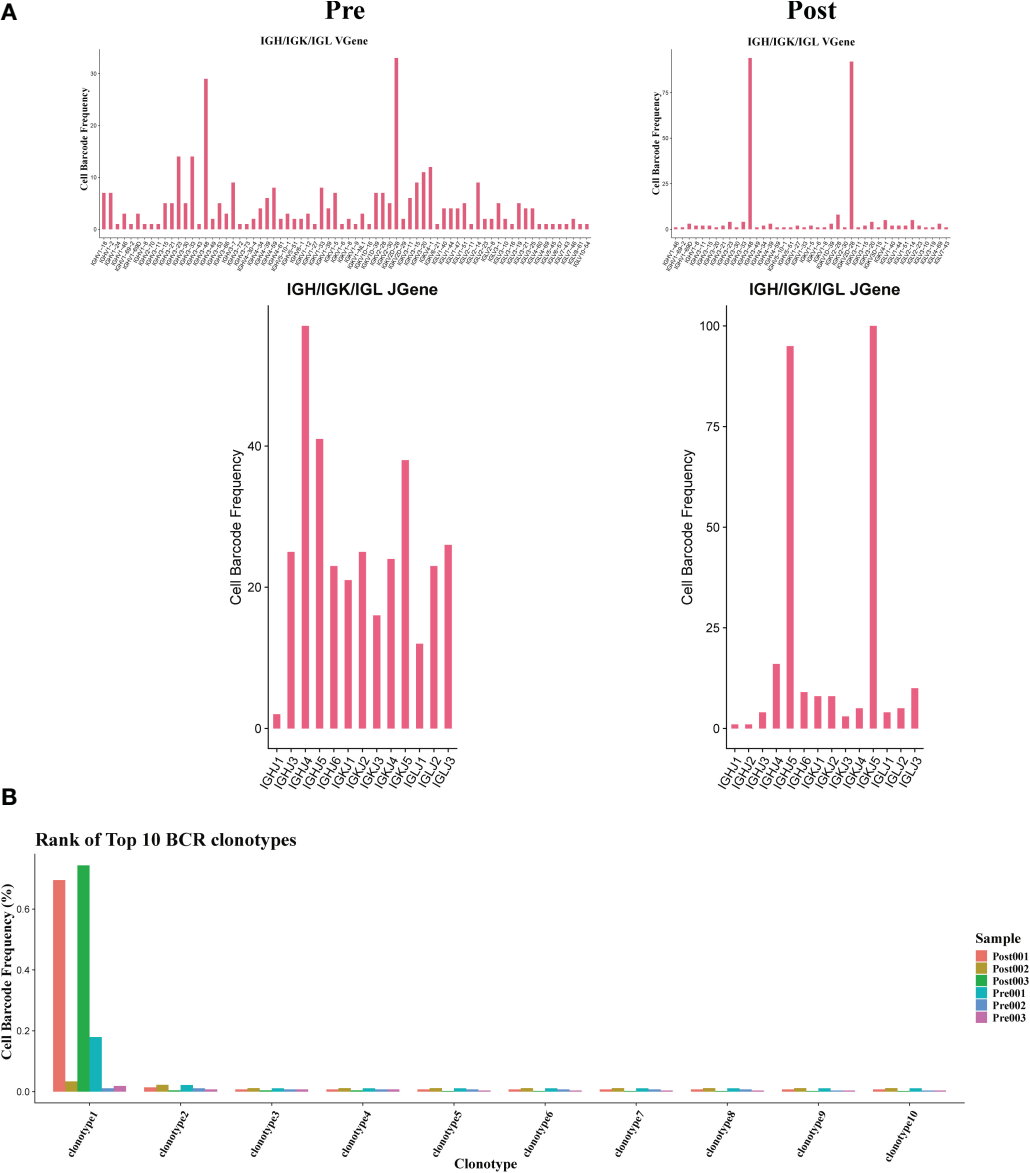
Usage of V(D)J genes and frequency of clonotypes in B cells. **(A)** The usage of the V(D)J gene of BCR in B cell between pre and post group. **(B)** The frequency of BCR clonotypes in B cells of different groups.

The results showed that the BCR clonotypes in donor liver was decreased after transplantation (**Figure 7B**). In addition, the usage of V and J genes in IGH/IGK/IGL chains was biased between the pre and post group. In BCR IGH, the most used J gene is IGHJ4 in pre group, while in post group it is IGHJ5 (**Figure 7A**). The overlapping of BCR among samples was shown in **Figure S1C**.

### 3.8 scRNA-seq of NK/NKT cell clustering and subtype analysis during liver transplantation

**Figure 8:**
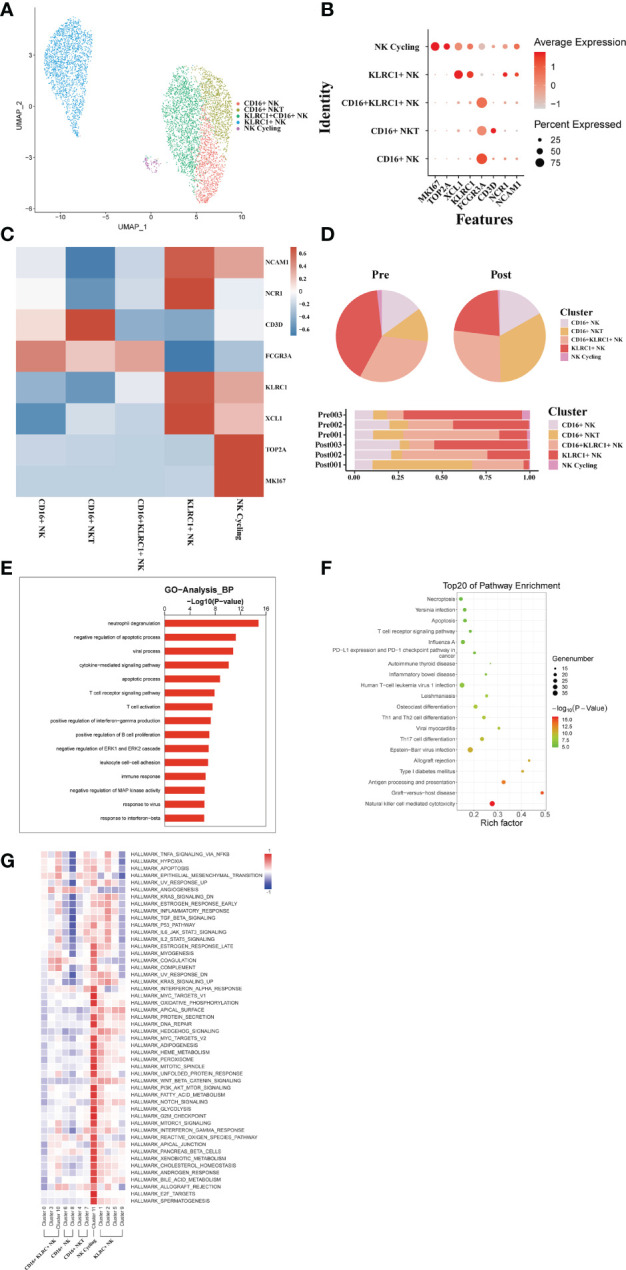
scRNA-seq of NK/NKT cell clustering and subtype analysis during liver transplantation. **(A)** UMAP plot of 5 different NK/NKT cell clusters during liver transplantation, colored according to diverse clusters. **(B)** Distinctive genes expression of FCGR3A, KLRC1, NCAM1,MKI67 and NCR1 for NK/NKT cell clusters. **(C)** Heatmap of 3 differentially expressed genes of each NK/NKT cell cluster. **(D)** The proportion of cells in different NK/NKT cell populations in each sample. **(E)** Gene Ontology enrichment analysis results of B/plasma cell clusters. **(F)** Result of KEGG pathway analysis showing the top 20 significantly different pathways activation in different samples of NK/NKT cell clusters. **(G)** Heatmap of hallmark pathways activation in different NK/NKT cell clusters.

From these three sets of donor liver samples, 6029 NK/NKT cells’ transcriptome data were gathered. We classified NK/NKT cells into five subsets based on the expression of characteristic NK/NKT cell markers to explore the dynamics of the various NK/NKT subtypes (**Figure 8A**). These clusters revealed one NK cycling cell (MKI67) subset clusters, one KLRC1+ NK cell (KLRC, XCL1) subset clusters, one CD16+ KLRC1+ NK cell (FCGR3A), one CD16+ NKT cell (KLRC1, CD3D), and one CD16+ NK cell (FCGR3A) clusters in the donor liver with different canonical marker genes (**Figures 8B, C**). Notably, compared to the pre-operative period, the number of CD16+ NKT cell subpopulations in the liver rose dramatically, whereas the proportion of KLRC1+ NK cell clusters dropped (**Figure 8D**). We used GO analysis to analyze the expression profiles of NK/NKT cells pre- and post-transplantation in order to further study the various transcriptome alterations in NK/NKT cells. We observed an enrichment of DEG in the *neutrophil degranulation* and *negative regulation of apoptotic process* (**Figure 8E**). And the pathway enrichment result showed that two pathways, *Graft−versus−host disease* and *allograft rejection* are associated with NK/NKT cells (**Figure 8F**). The results of QuSAGE analysis showed that the expression of genes in the cycling NK cell and KLRC+ NK cell clusters related to *IL6 JAK STAT3 signaling and IL2 STAT5 signal*, while the CD16+ NK cell clusters were inhibited in *those* pathways (**Figure 8G**).

## 4 Discussion

Some reports applied scRNA-seq technique to profile differences in the distribution of immune cells and transcriptome heterogeneity in solid organ transplants(33, 35). During liver transplantation, immune cells from the donor graft will enter the recipient patient and reshape the immune microenvironment together with the recipient’s own immune cells. However, there are no comprehensive research that has fully revealed how this immune shift affect IRI, immune rejection, immune tolerance and GVHD involving with organ transplantation. In this study, we annotated a comprehensive transcriptome profile of intrahepatic monocytes/Kupffer cells, NK cells, T cells, B cells and plasma cell clusters, and resolved a more detailed immunological response landscape.

Studies have reported a landscape of human liver cells using scRNA-seq and observed the presence of macrophages with different functions in normal liver tissue(33, 34). Similarly, our study has classified, annotated various heterogeneous immune cell clusters, and then analyzed the different biological functions of them in the liver. The difference is that our study focuses more on resolving the dynamics of the transcriptome of different cell clusters in the donor liver during transplantation, and carries out a customized bioinformatics analysis. In addition, immune repertoire study was carried out to obtain a more comprehensive immunological information. A study has used MARCO as a marker gene to identify the anti-inflammatory and pro-inflammatory phenotypes of macrophages(34). This was also used in our liver transplant scRNA-seq dataset to study macrophages with different anti-inflammatory and pro-inflammatory effects in the donor liver.

The inflammatory cascade triggered by macrophages is the critical mechanism leading to liver damage in IRI(36, 37). After reperfusion, the accumulated damage associated molecular patterns (DAMPs) and inflammatory mediators are released into the liver, activating Kupffer cells and producing more ROS, TNF-α, IL-1β and other pro-inflammatory cytokines(38). At the same time, inflammatory cytokines lead to the recruitment of monocytes, neutrophils, and T cells from the periphery, creating inflammatory cascade. On the other hand, some reports also claimed that Kupffer cells can reduce hepatic IRI damage by producing IL-10 production(39). In the present study, we found that functionally distinct clusters of Kupffer cells do exist in the donor liver, which is consistent with previous studies, and confirmed the dual role of Kupffer cells in liver IRI. In our results, such as the NF-κB and other inflammatory related pathways were activated after reperfusion in tissue macrophage clusters. This indicates that macrophages from liver play a more important pro-inflammatory role in the early stage of reperfusion.

T cells and B cells are the cell populations most closely associated with transplantation-related rejection, immune tolerance and GVHD. We found an increased proportion of CD4+Tem and CD8+Tem/Temra cell clusters in postoperative grafts, suggesting that there are more resident memory T (Trm) cells in liver grafts. Trm plays an immunomodulatory role in several organs(40). Trm also serves in the coordination of the adaptive immune system through crosstalk(41). By annotating the different immune cell types in liver transplantation, we assessed the relative importance of different immune checkpoint molecules in human liver transplantation. These molecules, such as CD44, LAG3, HAVCR2, BTLA, and PDCD1, are highly expressed in CD8+ T cells. At the same time, our data also suggested that some T cell populations in the transplanted liver are decreasing.

The diversity and composition of the TCR/BCR repertoire is the result of recombination of the V, (D), and J genes in the TCR/BCR gene locus, which has been found to be associated with T/B cell responses in several diseases. In this study, we comprehensively profiled the T and B cell repertoire in liver transplantation organs by single-cell combined TCR/BCR repertoire sequencing. T cells in the donor organ have fewer overlapping clones compared to pre-transplant donor T cells. This is the first comprehensive study to establish the association between T cell activation and liver transplantation at the level of the TCR and BCR clones. In addition, identification of TCR/BCR repertoire can identify antibodies that contribute to a functional immune response(42). TCR/BCR repertoire can also be used as diagnostic biomarkers or therapeutic targets for GVHD. The immune repertoire sequencing-based approach may represent a new personalized strategy to guide anti-rejection and induction of immune tolerance strategies after liver transplantation.

There are some limitations in our study, firstly we did not obtain enough hepatocytes which play a very important role in liver transplantation. The hepatocyte population is especially vulnerable to damage. Second, we need a larger sample size to understand the dynamics of the cells for more detailed understanding.

In summary, our study first annotated differences in subpopulations of multiple immune cell types in the donor graft during the liver transplantation, and explored their transcriptional features, which allowed us to better understand the reshaping mechanisms of the immune microenvironment. In addition, using immune repertoire sequencing technology, we have successfully studied the entire diversity of the immune pool at the single cell level. It can help us to accurately monitor immunosuppressive strategies and has the potential to identify new therapeutics.

## Data availability statement

The raw data presented in this paper have been deposited in online repositories. The name of the repository and accession number can be found below: NGDC Genome Sequence Archive (https://ngdc.cncb.ac.cn/gsa-human/); HRA003896.

## Ethics statement

The studies involving human participants were reviewed and approved by the Ethics Committee of Ruijin Hospital affiliated to Shanghai Jiao Tong University School of Medicine. The patients/participants provided their written informed consent to participate in this study.

## Author contributions

SS, YS, and ZF designed the study; LZ, SS, and HL collected the clinical data and liver sample; YS, LW, and TL contributed to the library and data generation; WL, DQ, and HB analyzed the data; SS, YS, and HB wrote the paper. All authors contributed to the article and approved the submitted version.

## References

[1] Rawal N Yazigi N. Pediatric liver transplantation. Pediatr Clin North Am (2017) 64:677–84. doi: 10.1016/j.pcl.2017.02.003 28502445

[2] Dar WA Sullivan E Bynon JS Eltzschig H Ju C. Ischaemia reperfusion injury in liver transplantation: Cellular and molecular mechanisms. Liver Int (2019) 39:788–801. doi: 10.1111/liv.14091 30843314 PMC6483869

[3] Briceno J Calleja R Hervas C. Artificial intelligence and liver transplantation: Looking for the best donor-recipient pairing. Hepatob Pancreat Dis Int (2022) 21:347–53. doi: 10.1016/j.hbpd.2022.03.001 35321836

[4] Lee BT Fiel MI Schiano TD. Antibody-mediated rejection of the liver allograft: An update and a clinico-pathological perspective. J Hepatol (2021) 75:1203–16. doi: 10.1016/j.jhep.2021.07.027 34343613

[5] Kitajima T Henry M Ivanics T Yeddula S Collins K Rizzari M. Incidence and risk factors for fatal graft-versus-host disease after liver transplantation. Transplantation (2021) 105:2571–8. doi: 10.1097/TP.0000000000003607 33449608

[6] Zhai Y Petrowsky H Hong JC Busuttil RW Kupiec-Weglinski JW. Ischaemia-reperfusion injury in liver transplantation–from bench to bedside. Nat Rev Gastroenterol Hepatol (2013) 10:79–89. doi: 10.1038/nrgastro.2012.225 23229329 PMC3577927

[7] Jadlowiec CC Taner T. Liver transplantation: Current status and challenges. World J Gastroenterol (2016) 22:4438–45. doi: 10.3748/wjg.v22.i18.4438 PMC4858627 27182155

[8] Newell LF Dunlap J Gatter K Bagby GC Press RD Cook RJ. Graft-versus-host disease after liver transplantation is associated with bone marrow failure, hemophagocytosis, and DNMT3A mutations. Am J Transplant (2021) 21:3894–906. doi: 10.1111/ajt.16635 33961341

[9] Gracia-Sancho J Villarreal G Jr. Zhang Y Yu JX Liu Y Tullius SG. Flow cessation triggers endothelial dysfunction during organ cold storage conditions: strategies for pharmacologic intervention. Transplantation (2010) 90:142–9. doi: 10.1097/TP.0b013e3181e228db PMC4522158 20606606

[10] Ingulli E. Mechanism of cellular rejection in transplantation. Pediatr Nephrol (2010) 25:61–74. doi: 10.1007/s00467-008-1020-x 21476231 PMC2778785

[11] Madill-Thomsen K Abouljoud M Bhati C Ciszek M Durlik M Feng S. The molecular diagnosis of rejection in liver transplant biopsies: First results of the INTERLIVER study. Am J Transplant (2020) 20:2156–72. doi: 10.1111/ajt.15828 32090446

[12] Kubes P Jenne C. Immune responses in the liver. Annu Rev Immunol (2018) 36:247–77. doi: 10.1146/annurev-immunol-051116-052415 29328785

[13] Travaglini KJ Nabhan AN Penland L Sinha R Gillich A Sit RV. A molecular cell atlas of the human lung from single-cell RNA sequencing. Nature (2020) 587:619–25. doi: 10.1038/s41586-020-2922-4 PMC7704697 33208946

[14] Papalexi E Satija R. Single-cell RNA sequencing to explore immune cell heterogeneity. Nat Rev Immunol (2018) 18:35–45. doi: 10.1038/nri.2017.76 28787399

[15] Krishna C DiNatale RG Kuo F Srivastava RM Vuong L Chowell D. Single-cell sequencing links multiregional immune landscapes and tissue-resident T cells in ccRCC to tumor topology and therapy efficacy. Cancer Cell (2021) 39:662–677.e666. doi: 10.1016/j.ccell.2021.03.007 33861994 PMC8268947

[16] Vallejo J Cochain C Zernecke A Ley K. Heterogeneity of immune cells in human atherosclerosis revealed by scRNA-seq. Cardiovasc Res (2021) 117:2537–43. doi: 10.1093/cvr/cvab260 PMC8921647 34343272

[17] Huang L Shi Y Gong B Jiang L Zhang Z Liu X. Dynamic blood single-cell immune responses in patients with COVID-19. Signal Transduct Target Ther (2021) 6:110. doi: 10.1038/s41392-021-00526-2 33677468 PMC7936231

[18] Schultheiss C Paschold L Simnica D Mohme M Willscher E von Wenserski L. Next-generation sequencing of T and b cell receptor repertoires from COVID-19 patients showed signatures associated with severity of disease. Immunity (2020) 53:442–455.e444. doi: 10.1016/j.immuni.2020.06.024 32668194 PMC7324317

[19] Liu X Zhang W Zhao M Fu L Liu L Wu J. T Cell receptor beta repertoires as novel diagnostic markers for systemic lupus erythematosus and rheumatoid arthritis. Ann Rheum Dis (2019) 78:1070–8. doi: 10.1136/annrheumdis-2019-215442 31101603

[20] Nakahara Y Matsutani T Igarashi Y Matsuo N Himuro H Saito H. Clinical significance of peripheral TCR and BCR repertoire diversity in EGFR/ALK wild-type NSCLC treated with anti-PD-1 antibody. Cancer Immunol Immunother (2021) 70:2881–92. doi: 10.1007/s00262-021-02900-z PMC10991438 33751180

[21] Degauque N Brouard S Soulillou JP. Cross-reactivity of TCR repertoire: Current concepts, challenges, and implication for allotransplantation. Front Immunol (2016) 7:89. doi: 10.3389/fimmu.2016.00089 27047489 PMC4805583

[22] Stuart T Butler A Hoffman P Hafemeister C Papalexi E Mauck WM 3rd. Comprehensive integration of single-cell data. Cell (2019) 177:1888–1902.e1821. doi: 10.1016/j.cell.2019.05.031 31178118 PMC6687398

[23] Zhang X Lan Y Xu J Quan F Zhao E Deng C. CellMarker: a manually curated resource of cell markers in human and mouse. Nucleic Acids Res (2019) 47:D721–8. doi: 10.1093/nar/gky900 PMC6323899 30289549

[24] Ramachandran P Dobie R Wilson-Kanamori JR Dora EF Henderson BEP Luu NT. Resolving the fibrotic niche of human liver cirrhosis at single-cell level. Nature (2019) 575:512–8. doi: 10.1038/s41586-019-1631-3 PMC6876711 31597160

[25] Sun Y Wu L Zhong Y Zhou K Hou Y Wang Z. Single-cell landscape of the ecosystem in early-relapse hepatocellular carcinoma. Cell (2021) 184:404–421.e416. doi: 10.1016/j.cell.2020.11.041 33357445

[26] Yaari G Bolen CR Thakar J Kleinstein SH. Quantitative set analysis for gene expression: a method to quantify gene set differential expression including gene-gene correlations. Nucleic Acids Res (2013) 41:e170. doi: 10.1093/nar/gkt660 23921631 PMC3794608

[27] Aibar S Gonzalez-Blas CB Moerman T Huynh-Thu VA Imrichova H Hulselmans G. SCENIC: single-cell regulatory network inference and clustering. Nat Methods (2017) 14:1083–6. doi: 10.1038/nmeth.4463 PMC5937676 28991892

[28] Yang HQ Wang YS Zhai K Tong ZH. Single-cell TCR sequencing reveals the dynamics of T cell repertoire profiling during pneumocystis infection. Front Microbiol (2021) 12:637500. doi: 10.3389/fmicb.2021.637500 33959105 PMC8093776

[29] Zheng C Zheng L Yoo JK Guo H Zhang Y Guo X. Landscape of infiltrating T cells in liver cancer revealed by single-cell sequencing. Cell (2017) 169:1342–1356.e1316. doi: 10.1016/j.cell.2017.05.035 28622514

[30] Crinier A Milpied P Escaliere B Piperoglou C Galluso J Balsamo A. High-dimensional single-cell analysis identifies organ-specific signatures and conserved NK cell subsets in humans and mice. Immunity (2018) 49:971–986.e975. doi: 10.1016/j.immuni.2018.09.009 30413361 PMC6269138

[31] Jourdan M Caraux A Caron G Robert N Fiol G Reme T. Characterization of a transitional preplasmablast population in the process of human b cell to plasma cell differentiation. J Immunol (2011) 187:3931–41. doi: 10.4049/jimmunol.1101230 21918187

[32] Xie X Shi Q Wu P Zhang X Kambara H Su J. Single-cell transcriptome profiling reveals neutrophil heterogeneity in homeostasis and infection. Nat Immunol (2020) 21:1119–33. doi: 10.1038/s41590-020-0736-z PMC7442692 32719519

[33] Wang L Li J He S Liu Y Chen H He S. Resolving the graft ischemia-reperfusion injury during liver transplantation at the single cell resolution. Cell Death Dis (2021) 12:589. doi: 10.1038/s41419-021-03878-3 34103479 PMC8187624

[34] MacParland SA Liu JC Ma XZ Innes BT Bartczak AM Gage BK. Single cell RNA sequencing of human liver reveals distinct intrahepatic macrophage populations. Nat Commun (2018) 9:4383. doi: 10.1038/s41467-018-06318-7 30348985 PMC6197289

[35] Li X Li S Wu B Xu Q Teng D Yang T. Landscape of immune cells heterogeneity in liver transplantation by single-cell RNA sequencing analysis. Front Immunol (2022) 13:890019. doi: 10.3389/fimmu.2022.890019 35619708 PMC9127089

[36] Abu-Amara M Yang SY Tapuria N Fuller B Davidson B Seifalian A. Liver ischemia/reperfusion injury: processes in inflammatory networks–a review. Liver Transpl (2010) 16:1016–32. doi: 10.1002/lt.22117 20818739

[37] Bao HL Chen CZ Ren CZ Sun KY Liu H Song SH. Polydatin ameliorates hepatic ischemia-reperfusion injury by modulating macrophage polarization. Hepatob Pancreat Dis Int (2022). doi: 10.1016/j.hbpd.2022.08.009 36058783

[38] Vardanian AJ Busuttil RW Kupiec-Weglinski JW. Molecular mediators of liver ischemia and reperfusion injury: a brief review. Mol Med (2008) 14:337–45. doi: 10.2119/2007-00134.Vardanian PMC2247470 18292799

[39] Bilzer M Roggel F Gerbes AL. Role of kupffer cells in host defense and liver disease. Liver Int (2006) 26:1175–86. doi: 10.1111/j.1478-3231.2006.01342.x 17105582

[40] Snyder ME Finlayson MO Connors TJ Dogra P Senda T Bush E. Generation and persistence of human tissue-resident memory T cells in lung transplantation. Sci Immunol (2019) 4. doi: 10.1126/sciimmunol.aav5581 PMC6435356 30850393

[41] Yuan R Yu J Jiao Z Li J Wu F Yan R. The roles of tissue-resident memory T cells in lung diseases. Front Immunol (2021) 12:710375. doi: 10.3389/fimmu.2021.710375 34707601 PMC8542931

[42] Dziubianau M Hecht J Kuchenbecker L Sattler A Stervbo U Rodelsperger C. TCR repertoire analysis by next generation sequencing allows complex differential diagnosis of T cell-related pathology. Am J Transplant (2013) 13:2842–54. doi: 10.1111/ajt.12431 24020931

