# Supplementary material for: Single-cell RNA-seq revealing the immune features of donor liver during liver transplantation
Source: Front Immunol. 2023 Feb 8;14:1096733. doi: 10.3389/fimmu.2023.1096733 (PMC9945228; doi:10.3389/fimmu.2023.1096733)
Supplement: Supplementary file 1 [file DataSheet_1.docx]

Supplementary Material


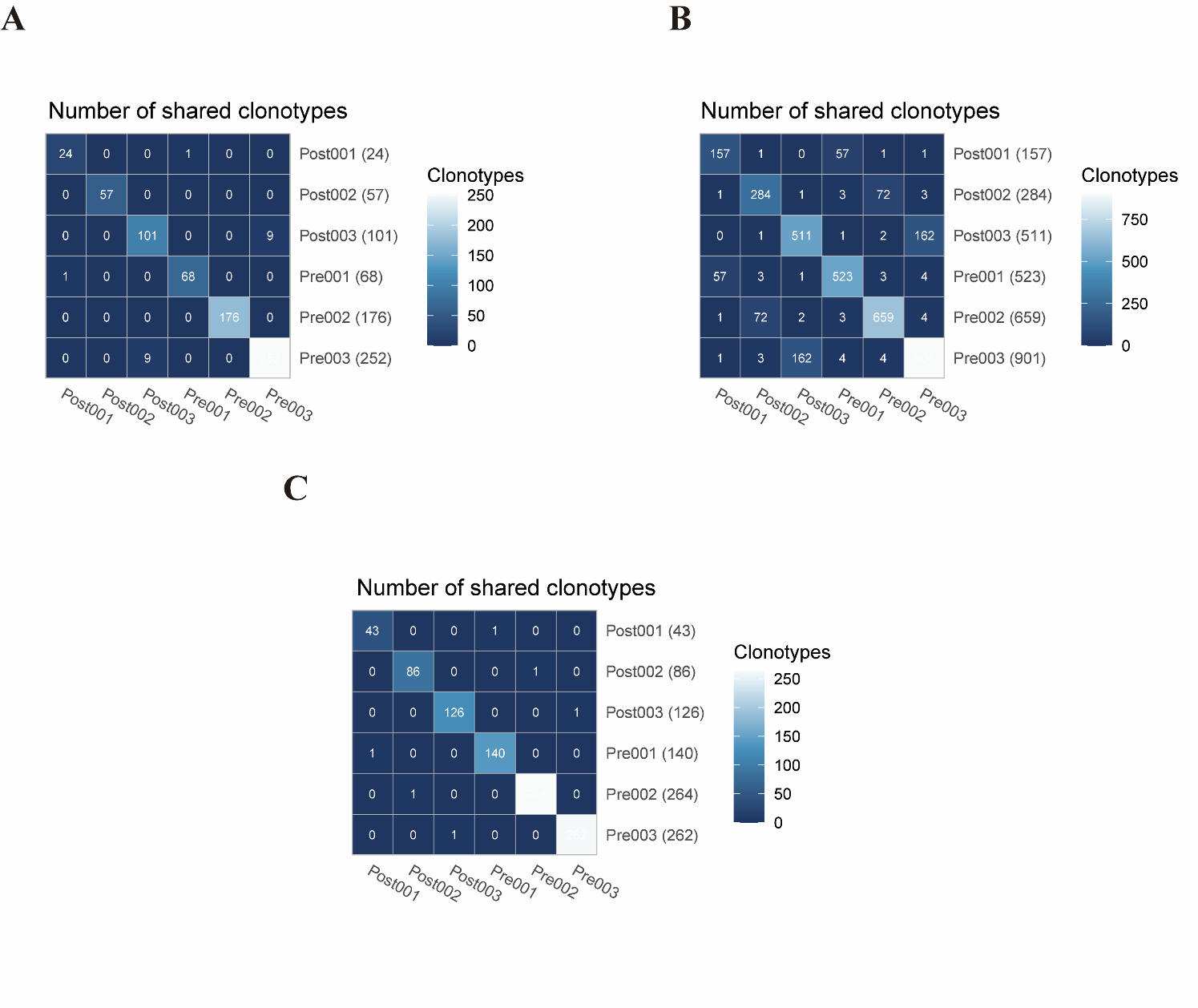


**Supplementary Figure 1.** The overlapping of TCR in CD4+T cell **(A)**, CD8+T cell **(B)** and BCR **(C)** among samples.
